# A Retrospective Study of Laboratory-Based Enteric Fever Surveillance, Pakistan, 2012–2014

**DOI:** 10.1093/infdis/jiy205

**Published:** 2018-07-27

**Authors:** Farah Naz Qamar, Mohammad Tahir Yousafzai, Shazia Sultana, Attaullah Baig, Sadia Shakoor, Farzeen Hirani, Abdul Wassay, Sehrish Khushboo, Junaid Mehmood, Alexander Freeman, Kashmira Date, Denise Garrett

**Affiliations:** 1 Department of Paediatrics & Child Health, Aga Khan University, Karachi, Pakistan; 2 Department of Pathology and Laboratory Medicine, Aga Khan University, Karachi, Pakistan; 3 Coalition Against Typhoid, Sabin Vaccine Institute, Washington, DC; 4 Global Immunization Division, Centers for Disease Control and Prevention, Atlanta, Georgia

**Keywords:** enteric fever, surveillance, South Asia, typhoid, paratyphoid, Pakistan

## Abstract

**Introduction:**

The Surveillance for Enteric Fever in Asia Project (SEAP) is a multisite surveillance study designed to capture morbidity and mortality burden of enteric fever (typhoid and paratyphoid) in Bangladesh, Nepal, and Pakistan. We aim to describe enteric fever disease burden, severity of illness, and antimicrobial resistance trends in Pakistan.

**Methods:**

In this retrospective, cross-sectional study, laboratory records of hospitalized patients who received a blood culture in any of 3 Aga Khan University hospitals in Karachi and Hyderabad, Pakistan, from 2012 to 2014 were reviewed. A case was defined as having a positive blood culture for *Salmonella* Typhi (*S.* Typhi) or *Salmonella* Paratyphi (*S.* Paratyphi). Antimicrobial sensitivity patterns were characterized for all *S*. Typhi and *S*. Paratyphi isolates. Medical records were available for abstraction (demographics, clinical features, complications) only among hospitalized cases.

**Results:**

Of the 133017 blood cultures completed during the study period, 2872 (2%) were positive—1979 (69%) for *S.* Typhi and 893 (31%) for *S.* Paratyphi. Fluoroquinolone resistance was present in >90% of both the *S.* Typhi and the *S.* Paratyphi isolates; almost none of the isolates were resistant to cephalosporins. Multidrug resistance (resistance to ampicillin, chloramphenicol, and cotrimoxazole) was observed in 1035 (52%) *S.* Typhi isolates and 14 (2%) *S.* Paratyphi isolates. Among *S*. Typhi and *S*. Paratyphi isolates, 666 (23%) were linked to hospitalized patients with medical records. Of the 537 hospitalized *S.* Typhi cases, 280 (52%) were aged 5–15 years, 133 (25%) were aged 2–4 years, 114 (21%) were aged >15 years, and 10 (2%) were aged 0–1 years. Among the 129 hospitalized *S.* Paratyphi cases, 73 (57%) were aged >15 years, 41 (32%) were aged 5–15 years, 13 (10%) were aged 2–4 years, and 2 (2%) were aged 0–1 years. Significant differences in symptomology between *S.* Typhi and *S.* Paratyphi cases were observed for nausea/vomiting, diarrhea, loss of appetite, and headache. Leukopenia, thrombocytopenia, and encephalopathy were the most commonly reported complications among enteric fever cases. No deaths were reported.

**Conclusion:**

Evidence of high antimicrobial resistance levels and disease severity support the need for continued surveillance and improved diagnostics for typhoid. Further prospective studies on vaccination as a tool for prevention of enteric fever in Pakistan are needed to inform disease intervention strategies.

## Teaser

2% of blood cultures performed at Aga Khan University Pakistan was positive for enteric fever during 2012–2014. Resistance to fluoroquinolone >90%, cephalosporin <0.1%, and multidrug resistance 52% (*Salmonella* Typhi) and 2% (*Salmonella* Paratyphi). Leukopenia, thrombocytopenia, and encephalopathy were common complications among hospitalized cases.

Globally, typhoid and paratyphoid fever (2 similar illnesses that constitute “enteric fever”) have been estimated to cause 190000 deaths annually [1]. More than 90% of enteric fever–related morbidity and mortality is reported from Asia, and studies from some Asian countries have shown that incidence of typhoid fever is highest among children aged <15 years [2, 3]. Because of a similar clinical presentation with other febrile illnesses, the utility of clinical diagnoses in enteric fever surveillance and disease burden estimation remains limited. Recent estimates of blood culture–confirmed cases are not available to calculate the incidence and disease severity of enteric fever in Pakistan [4]. Additionally, the rise in antibiotic-resistant strains of *Salmonella* Typhi (*S*. Typhi) and *Salmonella* Paratyphi (*S*. Paratyphi) has been associated with increased morbidity, mortality, and treatment costs [2, 3, 5, 6].

Existing studies have not provided reliable or recent population-based estimates to understand enteric fever disease burden and severity in South Asia. Data gathered from small-scale studies, such as hospital case reviews, are often focus on disease incidence in urban areas and are prone to spectrum bias focusing on severe cases, which has limited generalizability [3]. In Karachi from 1999 to 2001, passive surveillance at 2 field sites was used to estimate the annual serological incidence rate of typhoid to be 710 per 100000, whereas the annual incidence rate for blood culture–confirmed cases was estimated at 170 per 100000 [4]. The absence of credible estimates of the burden and severity of enteric fever has limited understanding of the impact of the disease and, therefore, hampers momentum for prevention and control efforts.

A substantial need remains to understand the changing enteric fever antimicrobial resistance trends in South Asia. Wong and colleagues describe a phylogeographical analysis that identified recent transfers and spread throughout Africa and Asia of a multidrug-resistant H58 lineage of *S.* Typhi [7]. Without additional understanding of the mechanism of drug resistance and development of strategies to control for resistance, treatment options for enteric fever are likely to become obsolete [8]. As part of the Surveillance for Enteric Fever in Asia Project (SEAP), this study aims to provide retrospective evidence on enteric fever disease burden, illness severity, and antimicrobial resistance trends in Pakistan.

## MATERIAL AND METHODS

### Study Sites

Hospitals in Karachi and Hyderabad, Pakistan, were assessed for study eligibility, which included having hospital laboratory blood culture capabilities and a systematic mechanism for capturing, archiving, and retrieving medical record data. Among the 8 hospitals assessed, 3 were eligible and participated in the study. Aga Khan University Hospital, located in Karachi, is a 700-bed tertiary-care hospital with approximately 61000 annual admissions in a catchment area of 23 million persons. Aga Khan Hospital for Women and Children, located in Karachi, is a 48-bed secondary-care hospital with approximately 2500 annual admissions in a catchment area of approximately 75000 persons. Aga Khan Hospital Hyderabad, located in Hyderabad, is an 87-bed secondary-care hospital with approximately 6500 annual admissions in a catchment are of approximately half a million persons. Both Aga Khan Hospital for Women and Children and Aga Khan Hospital Hyderabad provide care for only women and children. All Aga Khan University hospitals have centralized electronic medical and laboratory record databases that use unique identification numbers. The hospital labs have standardized blood culture techniques that use an automated culture system (culture contamination rate: <2%).

### Case Selection and Data Abstraction

The laboratory database was queried to identify all records with a blood culture positive for *S.* Typhi or *S.* Paratyphi from January 2012 to December 2014. For reporting of trends, all positive blood isolates were included; multiple positive laboratory records obtained from a single patient were excluded. Sensitivity testing at the lab follows Clinical and Laboratory Standards Institute guidelines. Antimicrobial resistance data patterns were abstracted, including those for ampicillin, chloramphenicol, cotrimoxazole, ciprofloxacin, cefixime, and ceftriaxone. Laboratory records were matched to medical records from hospitalized patients using unique identification numbers. Data abstracted from medical records included (1) demographics, (2) clinical presentation, (3) disease complications, (4) clinical investigations, and (5) diagnosis and patient outcomes.

### Data Management and Analysis

Data were collected on tablets with standardized forms and transferred daily to a server. A 10% quality control of all study records was conducted. The descriptive data analysis used SPSS version 20.0 (Armonk, New York). Multidrug resistance was defined as resistance to ampicillin, chloramphenicol, and cotrimoxazole [9]. Proportions of symptoms at the time of presentation were compared between patients with *S*. Typhi and patients with *S*. Paratyphi using Pearson χ^2^ tests. *P* values less than .05 were considered statistically significant.

### Ethical Considerations

The study was approved as nonresearch and exempted from a full ethical review by the Ethical Review Committee of the Aga Khan University, Karachi, Pakistan.

## RESULTS

From among the 133017 blood cultures available from lab records at the study sites from 2012 to 2014, 2872 (2%) were positive for enteric fever, including 1979 (69%) that were positive for *S.* Typhi and 893 (31%) that were positive for *S.* Paratyphi. The percentage of total culture-positive enteric fever cases that were *S.* Typhi increased from 67% (n = 584) in 2012 to 68% (n = 654) in 2013 and 72% (n = 690) in 2014.

There was a decreasing trend of resistance to ampicillin, chloramphenicol, and cotrimaxazole from 2012 to 2014 (Table 1). Resistance to ciprofloxacin was 93% in 2012, which slightly decreased to 90% in 2014. Although resistance to ceftriaxone was extremely low in our study population, there was a slight increase from 0.1% in 2013 to 0.3% in 2014 (Table 1).

**Table 1.** Proportion of *Salmonella* Typhi and *Salmonella* Paratyphi Isolates Resistant to Various Antibiotics From Aga Khan University Hospitals, Karachi and Hyderabad, Pakistan, by Year and Overall, 2012–2014

	Ampicillin	Chloramphenicol	Ciprofloxacin	Ceftriaxone	Cotrimaxazole	MDR
Year	No. (%)	No. (%)	No. (%)	No. (%)	No. (%)	No. (%)
*Salmonella* Typhi^a^						
2012	375/584 (64)	384/585 (66)	549/586 (94)	0/585 (0)	385/586 (66)	365/584 (63)
2013	339/654 (52)	355/681 (52)	602/677 (89)	1/667 (0.1)	359/681 (53)	332/675 (49)
2014	344/689 (50)	350/706 (50)	626/707 (86)	3/706 (0.4)	356/708 (50)	338/702 (48)
Total	1058/1927 (55)	1089/1972 (55)	1777/1970 (90)	4/1958 (0.2)	1100/1975 (56)	1035/1961 (53)
*Salmonella* Paratyphi						
2012	8/294 (3)	7/302 (2)	281/303 (93)	0/301 (0)	7/302 (2)	4/301 (1)
2013	10/304 (3)	10/314 (3)	290/313 (93)	0/308 (0)	12/314 (4)	5/311 (2)
2014	6/273 (2)	8/274 (3)	256/276 (93)	0/275 (0)	8/274 (3)	5/274 (2)
Total	24/871 (3)	25/890 (3)	827/892 (93)	0/884 (0)	27/890 (3)	14/886 (2)

Abbreviation: MDR, multidrug resistance (resistant to ampicillin, chloramphenicol, and cotrimoxazole).

^a^A total of 4 (0.2%) *Salmonella* Typhi isolates were resistant to cefixime, including 1 (<0.1%) in 2012, 1 (<0.1%) in 2013, and 2 (0.1%) in 2014.

**Figure 1.**
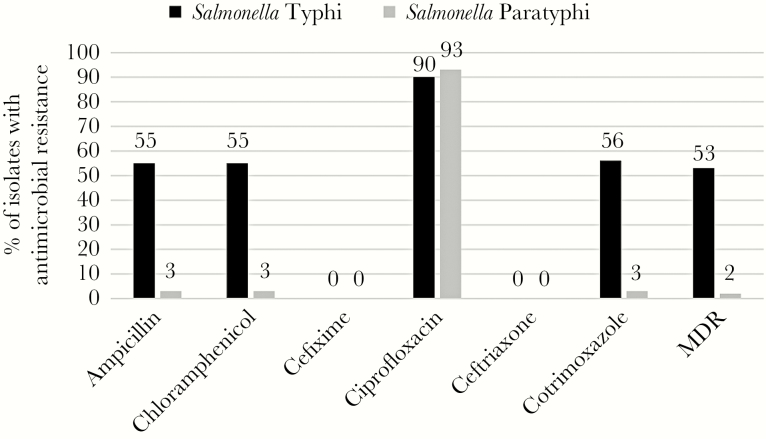
Antimicrobial resistance patterns among *Salmonella* Typhi and *Salmonella* Paratyphi isolates at Aga Khan University Hospitals, Karachi and Hyderabad, Pakistan, 2012–2014. Abbreviation: MDR, multidrug resistance.

**Figure 2.**
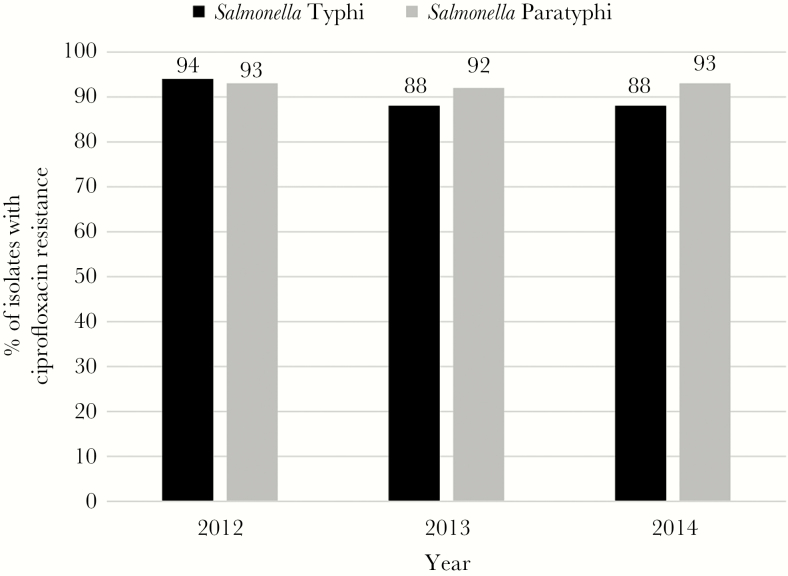
Ciprofloxacin resistance among *Salmonella* Typhi and *Salmonella* Paratyphi isolates at Aga Khan University Hospitals, by year, Karachi and Hyderabad, Pakistan, 2012–2014.

During the study period, elevated antimicrobial resistance was identified in *S.* Typhi isolates against ampicillin (55%), chloramphenicol (55%), and cotrimoxazole (56%); however, minimal resistance to all 3 antibiotics (≤3%) was observed in *S.* Paratyphi isolates (Figure 1, Table 1). Enteric fever isolates were almost entirely (>99%) sensitive to cephalosporins. Although resistance to ciprofloxacin has remained relatively stable at approximately 93% among *S.* Paratyphi isolates, a slight decrease in resistance has been observed from 94% to 88% in *S.* Typhi isolates from 2012 to 2014 (Figure 2). A decrease in multidrug resistance was observed from 2012 to 2014 and was driven by reduction in the resistance to ampicillin, chloramphenicol, and cotrimaxazole among *S*. Typhi isolates.

Among all enteric fever blood culture–positive cases, 2206 (77%) were outpatients and 666 (23%) were hospitalized. Most (55%) hospitalized enteric fever cases received care in pediatric wards (Table 2). A provisional diagnosis of enteric fever was provided to 86% of blood culture–confirmed enteric fever cases. Among the 537 hospitalized cases of *S.* Typhi, 353 (66%) were males. Similarly, males accounted for 58% of the 129 hospitalized *S.* Paratyphi cases. Comparable percentages were observed for cases among infants aged ≤1 year for *S.* Typhi (1.9%) and *S.* Paratyphi (1.6%). However, *S*. Typhi case patients were generally younger, with 79% aged ≤15 years compared with 43% of *S*. Paratyphi cases. Among enteric fever cases, the most commonly reported symptoms were fever (97%), nausea/vomiting (40%), and diarrhea (26%). *Salmonella* Typhi cases were more likely to have reported nausea/vomiting, diarrhea, and anorexia compared with *S*. Paratyphi cases (*P* < .01). *Salmonella* Paratyphi cases were more likely to have reported a headache (*P* < .05). Leukopenia was the most common complication, affecting 11% of *S*. Typhi cases and 16% of *S*. Paratyphi cases. Two cases of intestinal perforation were observed, 1 for each *S*. Typhi and *S*. Paratyphi. Encephalopathy was observed in 5 (0.9%) *S*. Typhi cases and 1 *S.* Paratyphi (0.8%) case. No deaths were recorded, and most cases of enteric fever were discharged, with <1% of cases requiring transfer to another hospital.

**Table 2.** Demographic and Clinical Characteristics of Patients Admitted With Blood Culture–Confirmed Enteric Fever at Aga Khan University Hospitals, Karachi and Hyderabad, Pakistan, 2012–2014

Characteristics of patients	Typhoid fever		Paratyphoid fever	
	No.	%	No.	%
No.	537		129	
Sex				
Male	353	65.7	75	58.1
Female	184	34.3	54	41.9
Age				
0–1 y	10	1.9	2	1.6
2–4 y	133	24.8	13	10.1
5–15 y	280	52.1	41	31.8
>15 y	114	21.2	73	56.6
Location of patient admission				
Medical	54	10.1	25	19.4
Pediatric	334	62.2	32	24.8
Surgical	12	2.2	12	9.3
Emergency	128	23.8	54	41.9
Other	9	1.7	6	4.7
Symptoms at the time of presentation				
Fever	522	97.2	121	93.8
Nausea/vomiting*	228	42.5	37	28.7
Diarrhea*	153	28.5	22	17.1
Abdominal pain	109	20.3	34	26.4
Cough/difficulty breathing	97	18.1	22	17.1
Loss of appetite/low intake*	102	19.0	10	7.8
General weakness	42	7.8	16	12.4
Headache**	32	6.0	14	10.9
Sore throat/flu	25	4.7	3	2.3
Seizures	19	3.5	3	2.3
Constipation	9	1.7	2	1.6
Other	82	15.3	26	20.2
Provisional diagnosis				
Enteric fever	462	86.5	108	83.7
Acute febrile illness	22	4.1	5	3.9
Pneumonia/RTI	17	3.2	5	3.9
UTI	6	1.1	0	0
Other	30	5.6	11	8.5
Complications				
Leukopenia^a^	61	11.4	20	15.5
Thrombocytopenia^b^	7	1.3	3	2.3
Encephalopathy	5	0.9	1	0.8
Hepatitis	3	0.6	1	0.8
Intestinal perforation	1	0.2	1	0.8
Hemodynamic shock	2	0.4	0	0
Renal impairment	1	0.2	0	0
Other	6	1.1	2	1.6
Outcome				
Sent home	505	94.0	121	93.8
Referred to another hospital	5	0.9	0	0
LAMA	18	3.4	5	3.9
Other	9	1.7	3	2.3

*Statistically significant result (*P* < .01) in the comparison between typhoid and paratyphoid patients.

**Statistically significant result (*P* < .05) in the comparison between typhoid and paratyphoid patients.

Abbreviations: LAMA, left against medical advice; RTI, respiratory tract infection; UTI, urinary tract infection.

^a^Leukopenia was defined as reduced white blood cell count (<5000 per microliter of blood). ^b^Thrombocytopenia was defined as reduced platelet count (<50000 per microliter of blood).

## DISCUSSION

This study provides critical information on burden, antimicrobial resistance trends, and illness severity of enteric fever in Pakistan from 2012 to 2014. The number of blood culture–confirmed cases reported in this study exceeds those reported in the same hospitals from 2009 to 2011 [10]. The highest proportion of typhoid cases were children aged 5–15 years, with more males than females affected. Our results are consistent with studies from Vietnam, where incidence of typhoid fever was highest among children aged 5–9 years [11] but diverge from some of the studies from India and Bangladesh that found the highest incidence of typhoid among children <5 years old [11].

The findings in our retrospective study raise several concerns about antimicrobial resistance; however, none are as alarming as the high prevalence of resistance to ciprofloxacin. Although no longer the standard of treatment for enteric fever in South Asia, fluoroquinolones (ciprofloxacin) continue to be preferentially prescribed in Pakistan. Without a rapid diagnostic test to guide physicians in the diagnosis of enteric fever, appropriate treatment of enteric fever will continue to be difficult, resulting in treatment failures. The standard treatments for enteric fever currently include cefixime and ceftriaxone [12]. Although a minimal level of resistance to cephalosporins was observed during the study period, antibiotic stewardship efforts will be imperative to retain the effectiveness of these disease treatment options. These concerns have already been realized in a recently reported outbreak of ceftriaxone-resistant *S*. Typhi in the Hyderabad city of Sindh, Pakistan, in 2017. During the first 6 months of the outbreak, >300 persons were identified by blood culture to be infected with *S*. Typhi resistant to first-line antibiotics (ampicillin, chloroamphenicol, and cotimaxazole) and ceftriaxone but sensitive to meropenem and azithromycin [8]. First-line antibiotics that make up the classification of multidrug resistance are no longer the choice of treatment for enteric fever. Decreased use of first-line drugs for enteric fever has likely contributed to the continued reduction in multidrug resistance in Pakistan [10].

Our results are consistent with a recent systematic review of enteric fever literature [13], which shows a high burden of disease in South Asia and finds that complications other than intestinal perforation are a frequent occurrence. A study conducted at a public-sector hospital in Pakistan reported 32 intestinal perforations over a 33-month period [14]. The prevalence of intestinal perforation is likely underestimated in this study because their inclusion criterion was a positive blood culture for an enteric fever pathogen. There are several reasons why patients with an intestinal perforation may not have a positive blood culture, such as failure to obtain a blood culture or presurgical antibiotic therapy prior to blood collection. In addition, because the study hospitals in this analysis are private, the cost of surgery may be prohibitive, resulting in intestinal perforation cases self-diverting to other hospitals for care. This study highlights that, although intestinal perforation is a hallmark complication of enteric fever, other complications, such as encephalopathy, are relatively common.

This retrospective study has several limitations; these will in part be addressed by a subsequent prospective SEAP study in Pakistan. First, the study design does not fully allow for disease burden to be estimated or incidence to be calculated. The disease burden in our study is underestimated due to absence of community because this was a hospital-based survey [12], the private hospital population is dissimilar to the community, only patients who had a blood culture were included in the study, and blood culture testing was used, which has a low sensitivity (40%–60%). Phylogenetic analysis was not conducted on any of the isolates reviewed in this study. Although the study provides evidence on retrospective antibiotic resistance trends, analysis to understand transmission patterns of *S*. Typhi with resistant genetic traits in Pakistan was unavailable. In particular, understanding the impact of *S*. Typhi H58 [15–17] and other resistant strains will be of future importance in maintaining effective treatment. Finally, the full spectrum of disease severity could not be captured because data were only abstracted from medical records. Future studies will need to assess long-term follow-up to capture all related disease complications and the associated social and economic impacts.

Evidence of high antimicrobial resistance levels and disease severity support the need for continued surveillance and improved diagnostics for typhoid. Further prospective studies on vaccination as a tool for prevention of enteric fever in Pakistan are needed to inform disease intervention strategies.

## Notes

***Financial support.*** This was supported by the Bill & Melinda Gates Foundation through Albert B. Sabin Vaccine Institute agreement no. 04-0234.

***Supplement sponsorship.*** This article is part of the supplement “Surveillance for Enteric Fever in Asia Project,” sponsored by the Sabin Vaccine Institute.

***Potential conflicts of interest.*** All authors: No reported conflicts of interest. All authors have submitted the ICMJE Form for Disclosure of Potential Conflicts of Interest. Conflicts that the editors consider relevant to the content of the manuscript have been disclosed.
